# A Rare Cardiac Cavernous Hemangioma Treated with Radiotherapy

**DOI:** 10.1155/2022/5698475

**Published:** 2022-09-05

**Authors:** Pule Wang, Daniel Chapman, Farzan Siddiqui

**Affiliations:** ^1^Wayne State University School of Medicine, 540 E Canfield St, Detroit, MI 48201, USA; ^2^Henry Ford Health System, 2800 W Grand Blvd, Detroit, MI 48202, USA

## Abstract

**Background:**

Although cardiac hemangiomas, as rare benign cardiac tumors, have been described in previous case reports, the role of radiation therapy in an unresectable cardiac hemangioma in adult has not been reported. We present a case report of a rare unresectable cardiac cavernous hemangioma treated with radiotherapy*. Case Presentation*. A 45-year-old female with new onset of coughing and worsening shortness of breath was found to have a biopsy proven cardiac cavernous hemangioma. Surgery was aborted due to excessive bleeding, and she was then treated with radiotherapy. A total dose of 30 Gy in 15 fractions was given using intensity-modulated radiation therapy (IMRT) to the mass with a modified 1 cm margin. Complete clinical symptomatic relief was achieved with reduction of the mass posttreatment. Ten-year follow-up revealed a stable, reduced hemangioma with no recurrence of symptoms.

**Conclusions:**

This is a rare example of cardiac hemangioma that developed in the right ventricle and compressed several major vessels. Radiotherapy may be safely used for treatment of unresectable cardiac hemangioma.

## 1. Introduction

Cardiac hemangiomas are extremely rare primary cardiac tumors, with a reported incidence of <2% among all primary heart tumors [1]. They can occur at any age and appear anywhere within the heart, with patients presenting with various clinical symptoms. Radiation therapy has been historically used to treat a broad variety of benign disorders. More clinical indications for radiotherapy at different anatomic sites including heart and peripheral vascular system have been identified and included according to international, up-to-date surveys [2]. However, treatment with radiation therapy has not been reported in any published cases for cardiac hemangioma in adults. Therefore, we report a rare case of surgically unresectable cardiac hemangioma that was treated successfully with radiation therapy. Ten years of follow-up has continued to show significantly reduced size followed by stability of the mass and no recurrence of clinical symptoms as of the last follow-up in 2021.

## 2. Case Presentation

**Figure 1:**
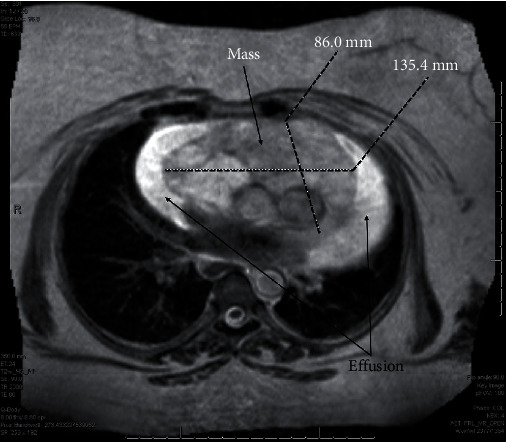
Magnetic resonance imaging at our institution revealed a lesion measuring 8.6 × 13.5 cm.

**Figure 2:**
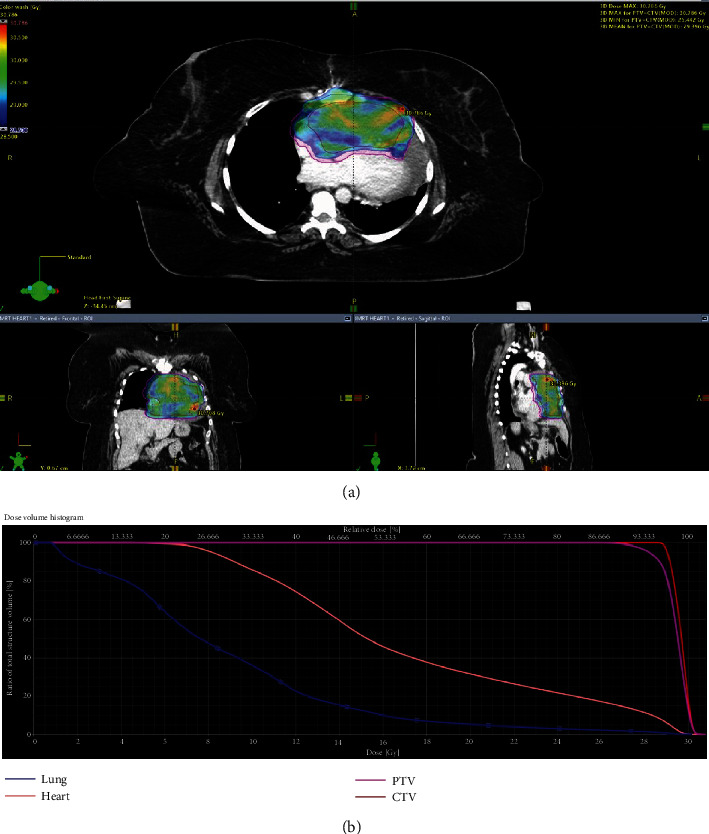
(a) IMRT radiation treatment plan and dose-volume histogram: clinical target volume (CTV) (red line): 95% of the dose going to 100% of the volume; planning target volume (PTV) (purple line): 95% of the dose going to 95% of the volume; heart (peach-colored line): mean heart dose of 17.2 Gy; lung (blue line): V20 = 5% and V5 = 75%. (b) Dose-volume histogram.

**Figure 3:**
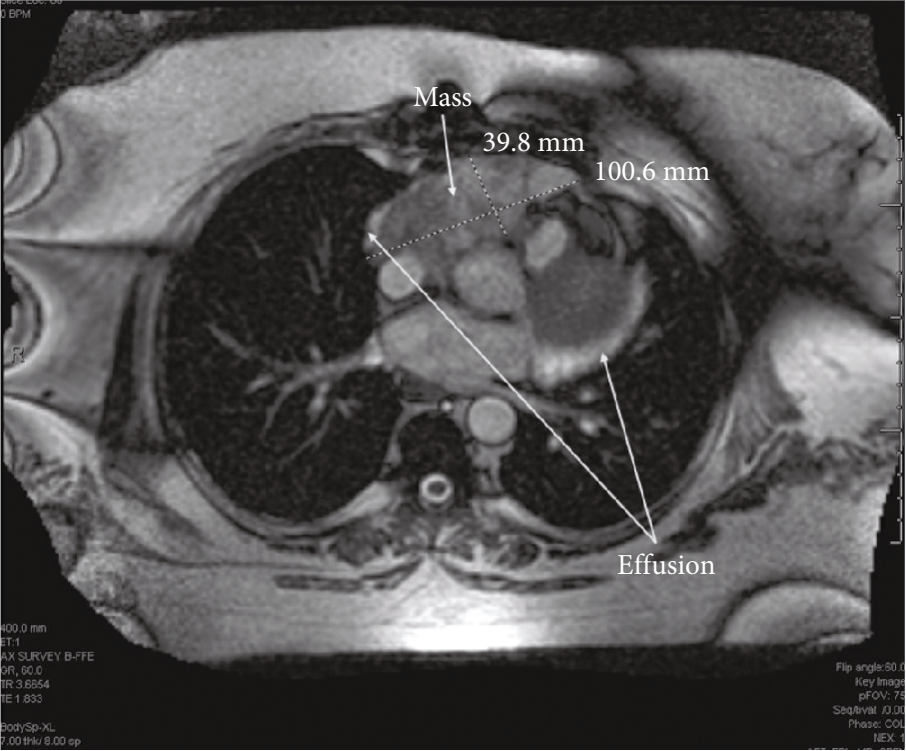
Follow-up magnetic resonance imaging demonstrated decreased size of the lesion posttreatment measuring 3.98 × 10.1 cm.

**Figure 4:**
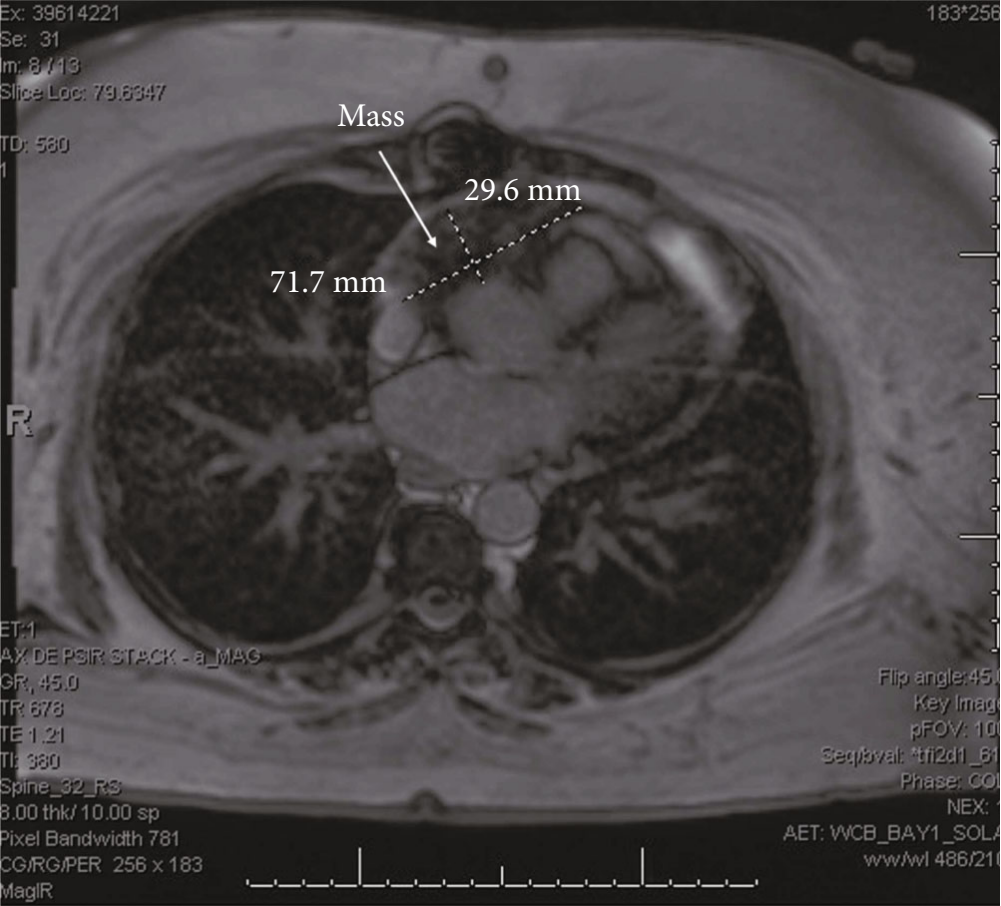
Follow-up magnetic resonance imaging demonstrated decreased size of the lesion posttreatment measuring 2.96 × 7.17 cm.

A 45-year-old female with years of excessive tiredness presented with coughing and worsening shortness of breath. Marked cardiomegaly with probable pericardial effusion was found on chest X-ray. This was followed by a CT angiogram of the chest, which showed an ill-defined mass extending from the pericardial surface anteriorly and pressing upon or involving the right ventricular free wall with moderate-to-large amount of pericardial effusion. A subsequent cardiac MRI showed a partly mobile intrapericardial mass anterior to the right atrium (RA) and right ventricle (RV) measuring 8.6 × 13.5 cm in size (Figure 1). Encasement of the main pulmonary artery and right coronary artery with partial encasement of ascending aorta, right superior pulmonary veins, and superior vena cava (SVC) was also noted. There was no gross invasion into these vessels. This was initially diagnosed as a liposarcoma, and cardiothoracic surgery was consulted for resection. Intraoperatively, the tumor was deemed to be unresectable as it was invading into the wall of the RV, and there was excessive bleeding. However, a biopsy was obtained and reported as a cavernous hemangioma. At this point, radiation oncology was consulted, and the decision was made to offer her external beam radiation therapy (EBRT). She received a course of 30 Gy in 15 fractions using intensity-modulated radiation therapy (IMRT) to the mass with a modified 1 cm margin in May of 2009. IMRT was chosen to allow precise radiation dose to the target and reduce dose to surrounding organs at risk (OAR) such as the heart, lungs, and esophagus. Patient was treated successfully with 6-MV photons delivered using a Varian Linear Accelerator (Varian Medical Systems Inc., Palo Alto, CA). The mean heart dose was 17.2 Gy, and the volume of the lung receiving 20 Gy was 5% (Figures 2(a) and 2(b)). This plan met (modified) quantitative analysis of normal tissue effects in the clinic (QUANTEC) dose constraints for both heart and lungs; mean heart dose is less than 26 Gy and bilateral lungs V20 less than 30% [3, 4]. A repeat cardiac MRI (Figure 3) a year after treatment showed a significant decrease in the size of the tumor and no reaccumulation of pericardial fluid. The patient was followed on a regular basis by the cardiologist and subsequently underwent regular cardiac MRIs over many years showing stable mass size without pericardial effusion; no symptom recurrence was ever noted. Patient was last seen by cardiology in 2021 and was doing well with no current complaints. The most up-to-date MRI as of June 2022 was read as continued decrease in size in the mass upon comparison to prior imaging (Figure 4).

## 3. Discussion

Cardiac hemangiomas are extremely rare primary cardiac tumors, with a reported incidence of <2% among all primary heart tumors [1]. They are benign vascular tumors composed of cavernous vascular channels or capillaries [5]. A capillary hemangioma is the most common type of hemangioma, and it is made of small capillaries that are normal in size and diameter. In contrast to a capillary hemangioma, a cavernous hemangioma in the heart is less common and is made up of multiple thin- and/or thick-walled vessels that are dilated. In our case, the tumor was a cavernous hemangioma. Most affected patients are asymptomatic, and majority of reported cases were identified while working up the symptoms. Among nonspecific symptoms, the most common one is exertional dyspnea [6–8]. In these studies, there is usually a variety of clinical presentations in patients depending on multiple factors including size and location. Our patient had a long history of shortness of breath, and she presented with acute onset of coughing and worsening shortness of breath.

Cardiac hemangiomas can arise anywhere and appear at any size in the heart. However, hemangiomas of the right ventricle with encasement of major vessels are extremely rare [7]. Among the 172 cases of cardiac hemangioma reviewed by Li et al., size varied from 0.5 to 14 cm with an average size of 4.48 cm. In this reported case, the size was measured to be 13.5 cm in greatest dimension. Only 7.6% exceeded 10 cm according to Li et al. Cardiac hemangiomas are usually misdiagnosed as other cardiac neoplasms. This is evidenced by the fact that more than 65% of cases, of a set of 44 reported, were misdiagnosed preoperatively [7]. It was not until proven by biopsy that the lesion in our patient was confirmed to be a cardiac cavernous hemangioma.

Surgical excision is the recommended first-line therapy in all symptomatic patients with cardiac tumors based on the low incidence of postoperative long-term adverse events and recurrence [9]. Partial tumor resection is equally as effective as complete removal [7]. Conservative treatment such as corticosteroid and radiotherapy for cardiac hemangioma was only reported in very few cases. Only one infantile case of an unresectable pericardial hemangioma treated with radiotherapy was reported by Yoshikawa et al. The pericardial hemangioma was resolved successfully in 2 months with delivery of 2,050 rad [10]. For treatment of unresectable large hemangioma of other bodily locations, radiation therapy has been reported to be effective in providing tumor shrinkage and pain relief in 13 cases. Doses greater than 25 or 30 Gy resulted in higher complete response with no long-term morbidity [11]. Alternatively, radiotherapy on vertebral hemangiomas has a great role in alleviating the symptoms of vertebral hemangiomas without compromising the quality of life [12, 13]. However, there is little information regarding radiation effectiveness and dose-response data in cardiac hemangioma. To be the best of our knowledge, this is the first report of a cardiac cavernous hemangioma treated with radiotherapy alone and having more than 10 years of follow-up. A total dose of 30 Gy was given with complete symptom relief after treatment. As such, it does seem reasonable that this data could be extrapolated to include hemangiomas of other bodily locations. Radiotherapy provided complete long-term (10 years) symptom relief which was clinically evident after the treatment as seen in our patient with a dose greater than 25 Gy. IMRT used in our patient is a feasible option in cardiac hemangiomas given its superior dose distribution and significant sparing of dosage to surrounding OARs. Recent advances in image-guided radiation therapy such as hybrid MR-linear accelerators may allow more efficient real-time tracking of the target and more efficacious sparing of OARs in benign conditions [2].

## 4. Conclusion

Surgically unresectable cardiac hemangiomas can be treated with radiation therapy alone with a dose of 30 Gy for symptomatic relief; however, further studies are needed to support our findings. Clinical symptoms and pericardial effusion both resolved in this case, and reduction in tumor size was also witnessed. As such, long-term outcomes seem successful and satisfactory.

## Data Availability

We prefer to deposit our data in a public repository that meets appropriate standards of archiving, citation, and curation. The findings of this paper should be publicly available whenever possible and as open as possible.
